# Acrodermatitis continua of Hallopeau: a review and update on biological and small molecule targeted immunomodulatory therapies

**DOI:** 10.3389/fimmu.2025.1525821

**Published:** 2025-08-15

**Authors:** Qiong Sun, Luyao Han, Zhimin Lin, Yuanhao Wu, Chen Li, Zhenhua Ying, Zuotao Zhao

**Affiliations:** ^1^ Department of Rheumatology and Immunology, Affiliated Hospital of Shaoxing University (Shaoxing Municipal Hospital), Zhejiang, China; ^2^ First Teaching Hospital of Tianjin University of Traditional Chinese Medicine, National Clinical Research Center for Chinese Medicine Acupuncture and Moxibustion, Tianjin, China; ^3^ Capital Medical University Beijing Hospital of Traditional Chinese Medicine, Beijing, China; ^4^ Department of Dermatology, Tianjin Institute of Integrative Dermatology, Tianjin Academy of Traditional Chinese Medicine Affiliated Hospital, Tianjin, China; ^5^ Department of Rheumatology and Immunology, Center for General Practice Medicine, Zhejiang Provincial People's Hospital (Affiliated People's Hospital, Hangzhou Medical College), Zhejiang, China; ^6^ Institute of Rheumatology and Immunology, Hangzhou Medical College, Zhejiang, China; ^7^ Zhejiang Provincial Key Laboratory of Traditional Chinese Medicine Cultivation for Arthritis Diagnosis and Treatment, Zhejiang, China

**Keywords:** Acrodermatitis continua of Hallopeau, immune targeted therapy, TNF inhibitor, IL-17 inhibitor, JAK inhibitor

## Abstract

Acrodermatitis continua of Hallopeau (ACH) is a rare aseptic pustular dermatosis for which clinical guidelines are lacking and treatment is largely based on case reports. Biologically targeted therapies offer new therapeutic ideas, with TNF antagonists such as adalimumab showing promising efficacy in both adults and children.The IL-17 and IL-23 axes play a key role in the pathogenesis of ACH, and anti-IL-17A and anti-IL-23 antibodies have shown therapeutic efficacy. In addition, new therapies such as IL-36 inhibitors and JAK inhibitors are being explored. Although biologics provide a new direction for ACH treatment, their safety and efficacy still need to be confirmed by large-scale clinical studies.

## 1 Introduction

**Figure 1:**
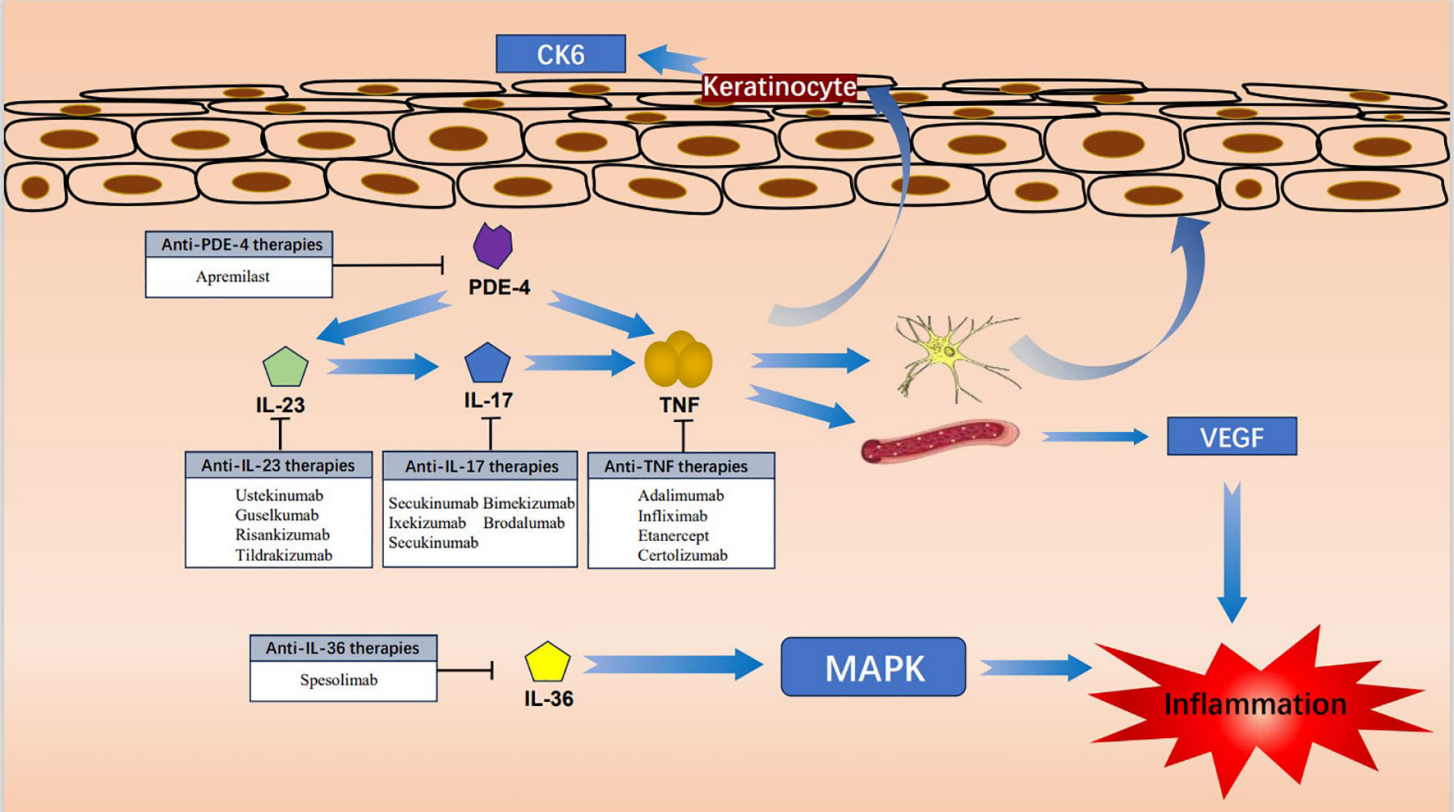
Diagram of the mechanism of pharmacological treatment of ACH.

Acrodermatitis continua of Hallopeau (ACH) is considered to be a subtype of pustular psoriasis that presents as a sterile, pustular eruption commonly in the fingertips and toes (1, 2). Given the rarity of ACH and its resistance to conventional treatments, there is a notable absence of established clinical guidelines for ACH. The current approach to treatment is largely informed by case reports. However, some patients who have not responded well to topical therapy and systemic therapy, have found relief with biologic therapy. This development offers a promising new avenue for therapeutic intervention in ACH. In the following we describe the current advances in clinical biologics and immune-targeted agents in the treatment of ACH, as well as the mechanism of action (**Figure 1**), and summarize the efficacy of currently published biologics and immune-targeted agents for the treatment of ACH (**Tables 1**, **2**).

**Table 1:** Summary of ACH cases treated with biological and small molecule targeted immunomodulatory therapies.

Age/Sex	Medicine	Patient characteristics	Dosage Schedule	Response to medication	Ref
12/male	Adalimumab	Multiple fingers of both hands affected	40mg every 2 weeks	Success; 4 weeks of treatment	(6)
23/female	Adalimumab	Multiple fingers of both hands affected, 10 years from onset of symptoms	80 mg at week 1, 40 mg at week 2 and 40 mg every 2 weeks	Success; 3 months of treatment	(6)
37/male	Adalimumab	Multiple fingers of both hands affected, 10 years from onset of symptoms	80 mg at week 1, 40 mg at week 2 and 40 mg every 2 weeks	Success; 10 weeks of treatment	(6)
14/female	Adalimumab	Multiple fingers of both hands and feet affected, 5 years from onset of symptoms	40mg every 2 weeks	Success; 3 months of treatment	(7)
68/male	Adalimumab	–	160 mg at week 0, followed by 80 mg at week 2, and 40 mg every week from week 4	Success; 2 months of treatment	(9)
70/female	Adalimumab	Multiple fingers of both Hands affected, 5 years from onset of symptoms	80 mg at week 1, followed by 40mg every 2 weeks	Success; 3 months of treatment	(10)
26/male	Adalimumab	Multiple fingers of both hands and feet affected, 4 months from onset of symptoms	40mg every 2 weeks	Success; 12 months of treatment	(11)
53/male	Adalimumab	1 finger affected, 10 years from onset of symptoms	40mg every 2 weeks	Success; 40 weeks of treatment Relapse after 28 weeks of discontinuation of the drug	(65)
62/female	Adalimumab	1 finger affected, 2 years from onset of symptoms	as above	Success; 3 months of treatment	(65)
54/female	Adalimumab	Nails on all fingers, on two toes and the soles of her feet, 4 months from onset of symptoms	40mg every week	Success; 1 month of treatment .	(66)
72/female	Adalimumab	Multiple fingers of both hands and feet affected, 1 year from onset of symptoms	40mg weekly at week1-7, followed by 40mg every 2 weeks	Success; 12 months of treatment, died because of metastatic lung cancer.	(67)
61/female	Adalimumab	Multiple fingers affected	40mg weekly at week1-44, followed by 40mg every 2 weeks	Success; 25 months of treatment	(67)
-/female	Adalimumab	Multiple fingers of both hands and feet affected	40mg every 2 weeks at week1-13, followed by 40mg week	Success; 24 months of treatment	(67)
72/female	Adalimumab	Multiple fingers of both hands and feet affected, 2 years from onset of symptoms	80 mg at week 1, 40 mg at week 2 and 40 mg every 2 weeks	Success; 9 months of treatment	(68)
79/male	Adalimumab	Third phalanx of the left foot affected, 2 years from onset of symptoms	80mg at Week 1, 40mg at Week 2, and 40mg every two weeks	Success; 1 month of treatment	(69)
20/female	Adalimumab	2 fingers affected, 2 years from onset of symptoms	40mg every 2 weeks	Success; 4 weeks of treatment	(70)
75/female	Adalimumab	Multiple fingers affected, 5 years from onset of symptoms	40mg every 2 weeks	Success; 4 weeks of treatment	(71)
15/female	Adalimumab biosimilar	Left foot affected, 6 months from onset of symptoms	40mg every 2 weeks	Success; 3 months of treatment	(8)
72/female	Infliximab	Multiple fingers of both hands and feet affected,15 years from onset of symptoms	5 mg/kg	Excellent response	(72)
78/female	Infliximab	Multiple fingers of both hands and feet affected, 7 years from onset of symptoms	5 mg/kg at 0, 2 and 6 weeks and maintenance doses at 8-week intervals	Excellent response High ANA after 40 months of treatment	(13)
60/female	Infliximab	Multiple fingers of both hands affected, 15 years from onset of symptoms	5 mg/kg at 0, 2 and 6 weeks and maintenance doses at 8-week intervals	Success; 32 months of treatment	(73)
50/female	Etanercept	Multiple fingers of both hands affected, 10 years from onset of symptoms	50mg weekly	Success; 6 months of treatment	(14)
8/male	Etanercept	Multiple fingers of both hands and feet affected, 8 months from onset of symptoms	25mg twice weekly	Success	(16)
8/female	Etanercept	Left first finger affected,8 months from onset of symptoms	25mg weekly	Success; 18 weeks of treatment	(17)
64/female	Etanercept	The thumb, the 2nd finger affected,43 years from onset of symptoms	25mg twice weekly	Success; 9 months of treatment	(18)
40/male	Etanercept	Multiple fingers of both hands affected	25mg twice weekly	Failure 12 weeks of treatment	(15)
29/female	Certolizumab Pegol	Multiple fingers of both hands and feet affected; 3 months from onset of symptoms	400 mg at weeks 0, 2, and 4 followed by 200 mg every 2 weeks,	Success; 10 months of treatment	(19)
23/female	Certolizumab Pegol	Multiple fingers of both hands and feet affected, 8 months from onset of symptoms	400 mg every 2 weeks for 1 month, 400 mg every month maintenance	Success; 18 months of treatment	(20)
43/male	Secukinumab	2 fingers and 1 toe affected, 1 years from onset of symptoms	300 mg at week 0,1,2,3 and 4, followed by 300 mg every 4 weeks	Success; 3 months of treatment	(24)
27/female	Secukinumab	1 finger affected, 1 year from onset of symptoms	as above	Success; 3 months of treatment	(23)
87/male	Secukinumab	Multiple fingers of both hands and feet affected, 2 years from onset of symptoms	as above	Success; 6 weeks of treatment .	(25)
42/female	Secukinumab	2 fingers affected, 3 years from onset of symptoms	5 subcutaneous weekly injections of 300 mg	Success; 1 month of treatment	(26)
25/male	Bimekizumab	2 fingers affected, 1 years from onset of symptoms	320 mg every 4 weeks	Success; 3 months of treatment	(74)
47/male	Brodalumab	Multiple fingers of both hands and feet affected, 1 year from onset of symptoms	210 mg at week 0,1, and 2, followed by 210 mg every 2 weeks	Success; 4 weeks of treatment	(29)
60/male	Brodalumab	1 finger affected, 20 years from onset of symptoms	as above	Success; 2 months of treatment	(22)
37/female	Brodalumab	Multiple fingers of both hands and feet affected, 3 years from onset of symptoms	as above	Success; 6 months of treatment	(30)
31/female	Ixekizumab	Multiple fingers of both hands and feet affected, 3 years from onset of symptoms	80mg every 2 weeks	Success; 10 weeks of treatment	(21)
72/male	Ixekizumab	1 finger affected, 2 years from onset of symptoms	Started at 160mg followed by 80 mg every 2 weeks	Success; 7 months of treatment	(28)
28/male	Ixekizumab	Multiple fingers of both hands affected, 2 years from onset of symptom	started 160 mg followed by 80 mg every 2 weeks from Week 2 through Week 12, and thereafter 80 mg every 4 weeks	Success; 6 months of treatment;	(75)
59/male	Ixekizumab	Multiple fingers of both hands and feet affected, 2 years from onset of symptoms	as above	Improved joints, no improvement in skin symptoms; 8 months of treatment	(76)
61/male	Ixekizumab	Multiple fingers of both hands affected, 6 years from onset of symptoms	–	Lack of improvement in skin symptoms; 4 months of treatment	(76)
61/male	Ustekinumab	1 finger affected, 1 year from onset of symptoms	45 mg at week 0,4, and 12, followed by 90 mg every 8 weeks	Rapid but short-lasting symptom improvement with 45mg treatment; Significant improvement in symptoms after 5 months of 90mg treatment	(42)
24/male	Ustekinumab	Multiple fingers of both hands affected, 13 years from onset of symptoms	45mg at week 0,4, then every 12 weeks	Success; 28 weeks of treatment	(37)
53/male	Ustekinumab	Multiple fingers of both hands affected, 1 year from onset of symptoms	45mg at week 0,4, then every 12 weeks	Dose needs to be increased to 90mg every 12 weeks in combination with acitretin for complete control	(39)
50/male	Ustekinumab	Multiple fingers of both hands and feet affected, 2 years from onset of symptoms	Started at 90 mg, followed by a 45 mg dose four weeks later, and then every 12 weeks thereafter	After treatment interruption, the retreatment with the ustekinumab plus acitretin (35 mg/day) was started again.	(35)
67/male	Ustekinumab	Multiple fingers of both hands affected, 8 years from onset of symptoms	45mg at week 0,4, then every 12 weeks	Success; 21 months of treatment	(40)
20/female	Ustekinumab	Multiple fingers of both hands affected	45mg at week 0,4, then every 12 weeks	Increase dose to 90mg every 12 weeks with complete symptom control	(41)
60/female	Guselkumab	Multiple fingers of both hands affected, 2 years from onset of symptoms	–	Success; 5 months of treatment	(77)
61/male	Guselkumab	Multiple fingers of both hands affected, 6 years from onset of symptoms	–	Success; 4 months of treatment	(77)
71/female	Guselkumab	Multiple fingers of both hands affected, 28 years from onset of symptoms	–	Success; 3 months of treatment	(77)
31/male	Guselkumab	1 finger affected, 1 years from onset of symptoms	1mg subcutaneous injection every two weeks	Success; 40 weeks of treatment	(43)
80/female	Risankizumab	Multiple fingers of both hands and feet affected, 20 years from onset of symptoms	150mg at weeks 0 and 4, and then every 12 weeks.	Success; 16 weeks of treatment	(45)
67/male	Risankizumab	Multiple fingers of both hands affected, 25 years from onset of symptoms	–	Success; 6 months of treatment	(77)
79/male	Risankizumab	Multiple fingers of both hands affected, 2 years from onset of symptoms	150mg at weeks 0 and 4, and then every 12 weeks.	Success; 4 months of treatment	(44)
62/male	Tildrakizumab	Multiple fingers of both hands and feet affected	–	Success; 21 months of treatment	(47)
9/female	Spesolimab	Multiple fingers of both hands and feet affected, 2 years from onset of symptoms	450 mg every 4 weeks	Success; 8 weeks of treatment	(50)
22/male	Spesolimab	Torso, multiple fingers of both hands and feet affected, 16 years from onset of symptoms	–	Success; 32 weeks of treatment	(78)
28/female	Baricitinib	Multiple fingers of both hands affected, 1 year from onset of symptoms	2mg/d	Success; 20 weeks of treatment	(54)
74/male	Deucravacitinib	Multiple fingers of both hands and feet affected, 2 years from onset of symptoms	6mg/q o d	Success; 24 weeks of treatment	(57)
72/male	Apremilast	1 finger affected	30/bid	Success; 16 weeks of treatment	(62)
62/male	Apremilast	Multiple fingers of both hands affected, 2 years from onset of symptoms	30/bid	Success; 3 months of treatment	(79)
61/female	Apremilast	Multiple fingers of both hands affected, 30 years from onset of symptoms	30/bid	Success; 12 weeks of treatment	(58)
58/male	Apremilast	Multiple fingers of both hands affected, 1 year from onset of symptoms	30/bid	Success; 4 weeks of treatment	(59)
75/male	Apremilast	Multiple fingers of both hands and feet affected, 1 year from onset of symptoms	30/bid	Success; 24 weeks of treatment	(61)

**Table 2:** Summary of the use of biologics and small molecule targeted drugs in ACH patients of different ages.

The age of patients	Biologics				Small molecule targeted drugs	
	TNFi	IL-17i	IL-(12)/23i	IL-36	Jaki	PDE-4i
<18 years of response						
Non-response*, n (%)	0%	0%	0%	0%	0%	0%
Partial response*, n (%)	20% (1/5)	0%	0%	0%	0%	0%
Excellent response*, n (%)	80% (4/5)	0%	0%	100% (1/1)	0%	0%
18–65 years of response^a^						
Non-response*, n (%)	7.1% (1/14)	0%	0%	0%	0%	0%
Partial response*, n (%)	21.4% (3/14)	18.2% (2/11)	11.1% (1/9)	0%	0%	0%
Excellent response*, n (%)	71.4% (10/14)	81.8% (9/11)	88.9% (8/9)	100% (1/1)	100% (1/1)	100% (3/3)
>65 years of response						
Non-response*, n (%)	0%	0%	0%	0%	0%	0%
Partial response*, n (%)	0%	0%	0%	0%	0%	0%
Excellent response*, n (%)	100% (8/8)	100% (2/2)	100% (5/5)	0%	100% (1/1)	100% (2/2)
Treatment duration (months), (median)	12^b^	7.5^c^	7.375	20	5.5	4
Patients with treatment duration ≥12 months, n (%)	58.8% (10/17)	0%	28.6% (4/14)	50% (1/2)	0%	20% (1/5)

^a^1 patient’ age is not mentioned.

^b^The treatment duration for 10 patients was not mentioned.

^c^The treatment duration for 3 patients was not mentioned. *One of the patients did not have a specific length of treatment.

## 2 TNF inhibitor

Recent studies have elucidated the role of tumor necrosis factor (TNF) in the inflammatory response. TNF is able to participate in the inflammatory response by promoting the expression of cytokeratin 6 (CK6) in keratinocytes, recruiting dendritic cells to the dermis (3), stimulating the formation of VEGF (4), and promoting the proliferation of keratinocytes (5), which are involved in the pathogenesis of psoriasis. Nowadays, TNF-α inhibitors have demonstrated promising clinical outcomes in the treatment of patients with ACH.

Adalimumab, a fully human monoclonal antibody, functions by inhibiting the interaction between TNF-α and its p55/p75 cell surface receptors. IL36RN mutations have been identified with increased prevalence in generalized pustular psoriasis (GPP). Furthermore, therapeutic interventions employing IL-17A antagonists have demonstrated efficacy in GPP. Preliminary research suggests that patients with ACH are more likely than patients with other forms of pustular psoriasis to carry two distinct mutations (ie, IL36RN and AP1S3 or IL36RN and CARD14) (5). ACH represents a distinct subtype of GPP, and it is possible for both conditions to coexist. A notable difference in cytokine expression has been observed between those affecting the nail bed and those impacting skin lesions, explaining the suboptimal response to IL-17A inhibitors but improvement in erythema and pustules in ACH patients with severe nail damage, who responded well after switching to adalimumab treatment (6). Several case reports have shown that adalimumab is effective in pediatric patients with ACH, and a case involving a pediatric patient with an IL-36RN mutation exhibited a substantial improvement in their lesions after switching to adalimumab following an unsatisfactory response to secukinumab (7). Additionally, adalimumab biosimilar has been reported for the treatment of ACH in children with sustained improvement (8), demonstrating the efficacy of adalimumab in a rare cohort of pediatric ACH patients. Significant improvements have also been seen in adult cohorts of patients with ACH combined with Crohn’s disease, rheumatoid arthritis, lung cancer, and psoriatic arthritis treated with adalimumab (9–11).

Infliximab is a monoclonal antibody with high specificity for TNF-α, reducing the inflammatory response and inhibiting hyperproliferation of keratinocytes. Although there are many reported cases with favorable results with infliximab, in some cases this treatment is limited by secondary loss of efficacy, positive antinuclear antibody titers, and infusion reactions (12). Infliximab has a greater probability of secondary loss of efficacy than adalimumab. In addition, elevated liver enzymes may occur with infliximab (13).

Etanercept is a fusion protein produced by recombinant DNA technology that targets and binds to soluble TNF-α in the serum, effectively neutralizing its pro-inflammatory effects and reducing its systemic levels. Clinical reports have documented successful treatment outcomes with etanercept in certain cases of ACH (14). However, there are also instances of poor response to etanercept therapy (15), which may correlate with the degree of progression of the patient’s disease. There are reports that children with ACH who developed resistance to infliximab have exhibited a positive response to etanercept. Furthermore, children with ACH and associated bone resorption had a remarkable efficacy of etanercept. These findings provide a compelling basis for the use of etanercept in the pediatric treatment of ACH (16–18). However, etanercept may also have the potential to cause elevated liver enzymes and should be monitored carefully when used.

Certolizumab is a polyethylene glycolated anti-TNF agent, characterized by minimal to negligible placental transfer, which makes it a low-risk therapeutic option for pregnant patients (19). There are case reports of ACH patients who responded well to guselkumab and continued to use guselkumab after successful delivery after switching to certolizumab at 12 weeks of gestation when lesions and pain scores remained significantly improved (20). Although certolizumab is classified by the FDA as acceptable for use in pregnancy, there are still studies reporting traces of the drug detected in neonates. Therefore, clinical benefits and potential risks need to be assessed before using biologics in pregnant patients, and when the clinical benefits surpass the risks, the safer certolizumab may be used.

## 3 IL-17 inhibitor

There are no clinical guidelines for ACH, but biologics targeting the psoriasis pathway have been shown to be effective against ACH. IL-17 is known to play a major role in the pathogenesis of psoriasis, and both anti-IL17A and anti-IL17RA antibodies have shown promising results in the treatment of psoriasis (21, 22). Therefore, target therapy for ACH patients can be realized by inhibiting IL-17.

Secukinumab, the first IL-17 antibody biologic approved for the treatment of plaque psoriasis, PsA, and ankylosing spondylitis, is a recombinant high-affinity human immunoglobulin G1κ monoclonal antibody. Several cases of successful treatment of ACH with secukinumab have been reported in clinical practice (23–26). And in some patients, it was found that TNFα therapy would lose its efficacy with prolonged treatment time, or need to be increased in dosage or in combination with other drugs, and after switching to secukinumab, significant results were achieved, so secukinumab may be an effective treatment option for patients with ACH who have failed conventional therapies.

Both ixekizumab and secukinumab are monoclonal antibodies that specifically inhibit IL-17 A. However, a recent meta-analysis showed that ixekizumab improved the psoriasis area and severity index (PASI) significantly more than secukinumab over a 12-week course of treatment, which provides new insight into the selection of an appropriate IL-17 inhibitor (27). The main reasons for choosing ixekizumab treatment today are its rapid onset of action, ability to treat concomitant joint symptoms, and safety in patients with underlying HBV infection (28).

Brodalumab, a fully human anti-IL-17 receptor A (IL-17RA) monoclonal antibody, has recently been successfully used in three patients with ACH (22, 29, 30). Brodalumab blocks not only the binding of IL-17A, but also its subtype IL-17F, which may explain why some patients respond to brodalumab but not to IL-17A-blocking antibodies.

Bimekizumab, a monoclonal IgG 1 antibody, has been found to be safe and effective in treating patients with moderate to severe plaque psoriasis (31). A case-control study found a 50%-70% improvement in nail involvement in patients with ACH and complete improvement in skin and joint symptoms in patients with SAPHO syndrome after treatment with bimekizumab. This suggests that bimekizumab may be a safe and effective treatment for patients with PPP, ACH, and SAPHO syndrome (32).

The list of potential therapies for the effective treatment of ACH is expanding with the growing number of biologics targeting IL-17, a cytokine. However, the mechanism of IL-17 treatment for ACH is still unclear, and more clinical data and experimental support are still needed.

## 4 IL-12/IL-23 inhibitor

Many scholars now believe that ACH is a subtype of pustular psoriasis, and that the immune system and the interleukin (IL)-17/IL-23 axis play key roles in the pathogenic mechanism, so the treatment of ACH is guided by its similarity to pustular psoriasis, and therefore IL-12/23 inhibitors and IL-23 inhibitors appear to be a promising option for the treatment of ACH (33, 34).

Ustekinumab, a monoclonal antibody against the shared p40 subunit of cytokines IL-12 and IL-23, has been used in more than 2,000 patients and has been found to be effective in treating moderate to severe psoriasis (35). In a multicenter retrospective study, ustekinumab showed improvement in 75.0% of patients with ACH (36). Some case reports suggest that ustekinumab is effective in the treatment of ACH, but complete regression of the lesions requires higher doses than in psoriasis vulgaris, often in combination with acitretin. As with other biologics, loss of response to ustekinumab can occur when treatment is interrupted, and reattainment of the therapeutic effect requires an increase in dosage or a combination of other medications (35, 37–39). However, in some case reports patients switched to ustekinumab in combination with acitretin after failing TNF inhibitor therapy and experienced rapid and sustained improvement in their condition (40–42). Indicates that treatment of ACH with ustekinumab may require high-dose maintenance therapy in combination with acitretin and methotrexate and may be an optional alternative therapy after TNF inhibitor therapy.

Guselkumab is also an IL-23 inhibitor, and a case of an ACH patient with single finger involvement whose rash continued to worsen after ixekizumab treatment was reported to have disappeared after switching to guselkumab microinjection therapy. This suggests that local injection of microdose biologics may be a precise and safe treatment for patients with limited ACH. Local injection of microdose biologics is more precise than systemic standard dose biologics, allowing antibodies to reach the target lesion more efficiently, resulting in lower costs and potential side effects (43). However, more clinical cases and clinical studies are still needed.

Risankizumab is a monoclonal antibody that selectively binds IL-23 cytokines and blocks their interaction with the IL-23 receptor. According to multiple case reports, risankizumab has shown good efficacy and safety in the treatment of elderly ACH patients, so we can prioritize risankizumab in elderly ACH patients (44, 45).

Tildrakizumab is a high affinity monoclonal antibody to humanized immunoglobulin IgG1/κ that specifically binds the IL-23p19 subunit. Global Phase III Clinical Trials (reSURFACE 1 and reSURFACE 2) demonstrate efficacy of tildrakizumab in patients with moderate to severe plaque psoriasis (46). And a case of successful treatment of an ACH patient with multiple comorbidities (advanced malignancy, recurrent pyothorax, psoriatic arthritis) using tildrakizumab has been reported (47), so tildrakizumab may be considered for ACH patients with malignancy or high risk of infection.

## 5 IL-36 inhibitor

A current clinical study in Taiwan suggests that approximately 50% of patients with interleukin-β 36 receptor antagonist (IL 36 RN) deficiency will exhibit symptoms of ACH, which provides pathophysiologic support for the use of IL-36 inhibitors in patients with ACH (48). In 2022, spesolimab, a monoclonal antibody targeting the IL-36 receptor, was approved for the treatment of GPP, and the safety and efficacy of spesolimab was evaluated in a phase I trial in seven patients with moderate GPP, with all seven patients showing a 70%-80% improvement and no significant adverse events in patients or healthy controls (49). Given the similarity between GPP and ACH, spesolimab has also been used in patients with ACH, and in this case, the patient experienced a rapid and significant response to the use of spesolimab (50). And current clinical trials suggest that GPP patients, with or without the IL36RN variant, may respond to spesolimab (51).

## 6 Jak inhibitor

The main Jak inhibitor currently used for the treatment of patients with ACH is baricitinib, an oral small molecule Janus kinase (JAK1) and (JAK2) inhibitor, which has good use in patients with atopic dermatitis (AD) (52). Baricitinib completely inhibits the JAK-STAT (signal transducer and activator of transcription) signaling pathway, thereby inhibiting targeted cytokines (e.g., TNF-α, IL-17, and IL-23), which could be a potential explanation for baricitinib treatment of ACH (53). A case has been reported in which a patient with ACH who had failed conventional treatment was switched to baricitinib with significant improvement in skin and joint symptoms and no new progression of bone erosion. This suggests that baricitinib is a potential treatment option for ACH and may inhibit the progression of bone disease (54).

TYK2, also a JAK family gene, plays a key role in mediating the signaling of multiple cytokines that cause inflammation. Deucravacitinib is a TYK2 inhibitor that inhibits TYK2-mediated inflammatory and immune responses by selectively inhibiting TYK2 activation and blocking signaling of inflammatory cytokines such as IL-23, IL-12, and type I interferon (IFN) (55). A phase III clinical trial in Japan demonstrated favorable clinical efficacy of deucravacitinib in patients with GPP, TYK2 inhibitor response in GPP may be facilitated by an unregulated IL-36 signaling pathway (56). Only one case has been reported of an elderly patient with ACH after treatment with smoking cessation, tonsillectomy, acitretin and cyclosporine failed, and after 24 weeks of treatment with deucravacitinib, the patient’s symptoms, including nail lesions, completely disappeared (57).

## 7 PDE-4 inhibitor

Apremilast, an oral phosphodiesterase 4 inhibitor, modulates pro-inflammatory pathways to promote anti-inflammatory activity, increases intracellular concentrations of cAMP, and blocks the production of pro-inflammatory cytokines, such as TNF-α, IFN-γ, IL-17, IL-23, and IL-12 (58, 59), which have been shown to be involved in the inflammatory response in systemic pustulosis and ACH (60). Apremilast is a relatively safe and easy to administer drug compared to other biologics. Based on the case reports reported to date, apremilast can be found to be a potential therapeutic option for ACH patients with comorbidities for biologics (e.g., tuberculosis, cancer, hepatitis B infection) and is also relatively safe and efficacious in elderly ACH patients with multiple comorbidities (58, 61, 62).

With further insights into the pathogenesis and treatment of psoriasis, most of the biologic therapies available for the treatment of plaque psoriasis have also shown significant efficacy in patients with ACH, even though some of the biologics require higher dosages and combination therapies, and still offer new therapeutic ideas for the treatment of ACH. However, with the emergence of new biologics and small molecule targeted drugs, the therapeutic choices for ACH patients will be more promising (63, 64). The number of cases reported in the literature is still small, most studies have small sample sizes, and the safety of biologics in treating ACH patients needs to be confirmed by large-scale clinical studies, and we look forward to further studies to confirm the efficacy of biologically targeted therapies in ACH patients.
